# Paternal and/or Maternal Blackberry (*Rubus* spp.) Polyphenolic Extract Consumption Improved Paternal Fertility and Differentially Affected Female Offspring Antioxidant Capacity and Metabolic Programming in a Mouse Model

**DOI:** 10.3390/antiox14070779

**Published:** 2025-06-25

**Authors:** Vanessa Cardoso Pires, Sara Lima Anacleto, Cristiane Matté, Odair Aguiar, Franco Maria Lajolo, Neuza Mariko Aymoto Hassimotto, Thomas Prates Ong

**Affiliations:** 1 Department of Food Science, School of Pharmaceutical Sciences, University of São Paulo, São Paulo 05508-000, Brazil; vanessacpires@usp.br (V.C.P.); sara.lima@usp.br (S.L.A.); fmlajolo@usp.br (F.M.L.); aymoto@usp.br (N.M.A.H.); 2 Department of Biochemistry, Institute of Fundamental Health Sciences, Federal University of Rio Grande do Sul, Porto Alegre 90610-264, Brazil; matte@ufrgs.br; 3 Department of Biosciences, Institute of Health and Society, Federal University of São Paulo, Santos 11050-020, Brazil; odair.junior@unifesp.br; 4 Food Research Center (FoRC), São Paulo 05508-000, Brazil

**Keywords:** blackberry, polyphenols, reproduction, POHaD, DOHaD, mice

## Abstract

Dietary polyphenols’ role in early life is not clear. While accumulating studies show both beneficial and deleterious effects of maternal consumption of these bioactive compounds on offspring’s adult health, very few studies have focused on the impact of paternal consumption. In addition, the potential interaction of combined parental polyphenol consumption is still not known. Thus, the aim of the present study was to investigate the effects of maternal (gestation/lactation) and/or paternal (preconception) blackberry polyphenol (anthocyanins, ellagitannins, and quercetin) methanolic extract consumption on C57BL/6 female mice offspring. Blackberry polyphenol consumption by fathers improved their sperm production and increased fertility. Blackberry polyphenol consumption by fathers, but not mothers, increased their plasma antioxidant capacity. All parental interventions decreased offspring perinatal mortality, with combined fathers’ and mothers’ polyphenol consumption exerting the most pronounced effects. Paternal or maternal polyphenol consumption decreased plasma total antioxidant capacity in the female offspring. On the other hand, combined parental consumption had opposing effects on the offspring. Only maternal polyphenol interventions increased glucose tolerance in the female offspring. These data only partially confirm our hypothesis that combined paternal and maternal polyphenol intervention would lead to better outcomes in the offspring. These results further show that blackberry polyphenols’ effects on offspring health depend on whether their consumption occurred through the father, mother, or both. This suggests that in order to promote long-term health in descendants, nutritional interventions, including those with polyphenols, should target not only the mother but also the future father.

## 1. Introduction

Blackberry (*Rubus* spp.) is considered an important source of polyphenols, including anthocyanins and ellagitannins [1]. There is evidence that blackberry and its polyphenolic compounds have a potential role in improving metabolic function and preventing chronic diseases [2]. Anthocyanins and ellagitannins have been shown to exert such effects through their redox modulating actions [2]. Dietary polyphenols’ actions on redox homeostasis are multifaceted, as they can directly scavenge reactive oxygen species (ROS), as well as modulate the expression/activity of antioxidant or ROS-producing enzymes [3]. In addition, high levels of these compounds can elicit reductive stress [4]. Most studies have interrogated dietary polyphenols’ health effects when provided to adult individuals or animals. However, the timing of exposure is a key aspect that can influence polyphenols’ biological effects [5]. Thus, it is key to also consider their effects in other developmental windows, particularly in early life [5,6].

The Developmental Origins of Health and Disease (DOHaD) concept posits that experiences in utero can profoundly influence health status and disease risk in adult life [7]. Most DOHaD studies have focused on maternal obesity and high-fat diet consumption during gestation on fetal development and metabolic disease programming later in life [8,9]. Maternal supplementation with dietary polyphenols has been proposed as a promising clinical strategy to counteract these metabolic programming factors and improve offspring health [6,10]. An increasing number of clinical and experimental studies have focused on this possibility [11,12]. Most consistent clinical data highlight preeclampsia, gestational diabetes, and fetal growth restriction as the main pregnancy disturbances that could be improved by dietary polyphenol consumption, although there is still no consensus on the validity of supplementation for mothers [13]. Ingested polyphenols by mothers have been shown to cross the placental barrier and be distributed to the fetus [14]. In this context, it is of utmost importance to evaluate polyphenols’ consumption efficacy and safety both for the mother and baby [13].

In addition to maternal experiences, accumulating evidence shows that paternal ones, such as obesity, malnutrition, and smoking, can also impact offspring health and disease status in adulthood [15]. This highlights paternal preconception as a valuable additional window of opportunity to prevent diseases in offspring through nutritional interventions [16,17]. However, a very limited number of Paternal Origins of Health and Disease (POHaD) studies have examined the potential of food bioactive compounds [17]. McPherson et al. [18] observed that consumption of a mix of micronutrients, lycopene, and green tea catechins by male rats ameliorated low-protein diet-induced deregulations in embryo development, as well as in the metabolism and pancreatic function of the adult offspring. Of note, fruit and vegetable consumption is positively correlated with sperm quality and male reproductive health [19]. Thus, the potential beneficial health impact of the consumption of these foods and their bioactive compounds should be better elucidated in the context of POHaD [6,17].

Although blackberry presents an interesting polyphenolic profile, few in vivo studies have evaluated its health benefits, especially in the DOHaD/POHaD context. While no studies have been conducted with blackberry fruit on male and female reproductive health, accumulating data have shown that its main polyphenolic constituents, cyanidin-3-glucoside and quercetin, exerted beneficial effects in this context, both from a paternal and maternal perspective [20,21,22,23]. However, regarding ellagitannins, another blackberry main component, no information is available in this regard. Furthermore, information on blackberry fruit or its components’ impacts in the developmental programming context is scarce. A recent experimental study [24] showed the inhibition of metabolic programming in mice offspring from fathers on a high-fat/high-sugar diet that also received supranutritional levels of cyanidin-3-glucoside during preconception. In addition, maternal consumption during gestation of a blackberry juice containing a low concentration of anthocyanins increased the brain length and cell density of the dentate gyrus in the offspring [25].

Thus, we designed this study to evaluate, in C57BL6 mice, the effects of paternal and/or maternal consumption, during preconception (males) or gestation/lactation, of a polyphenol-rich (cyanidin, quercetin, and ellagitannins) blackberry methanolic extract, which provides supranutritional polyphenolic levels, on the fathers’ and mothers’ fertility, as well as on female offspring developmental, antioxidant, and metabolic parameters. Our hypothesis was that all blackberry interventions would elicit beneficial effects on female offspring health regarding these parameters, with combined paternal and maternal interventions leading to the most pronounced effects. To the best of our knowledge, this is the first blackberry study with a combined DOHaD/POHaD approach. Among its strengths, we highlight the fact that such an experimental design allowed the comparison between maternal and paternal effects, as well as their interaction.

## 2. Materials and Methods

### 2.1. Blackberry Methanolic Extract Preparation

Blackberry fruit (*Rubus* spp.) was acquired from Sítio do Bello farm (Paraibuna, Brazil) and kept frozen (−20 °C) until the preparation of the methanolic extract for administration to the animals. It was prepared by milling frozen fruit samples in 70% methanol acidified with 0.2% acetic acid (1:5 proportion), grinding it for 30 s at an intermediate speed, with subsequent filtration by gravity using filter paper. After that, the extract was concentrated through the removal of methanol under a vacuum at 40 °C on a rotary evaporator (Rotavapor RE 120; Buchi, Flawil, Switzerland), then diluted in ultrapure water (10 mL) and stored at −80 °C. The blackberry methanolic extract was prepared every week to guarantee the phenolic content offered to the animals. A stability test was performed to quantify the phenolic compounds in the methanolic extract stored at −80 °C for 7 days. The antioxidant capacity, total phenolic content, flavonoids profile and content, and ellagitannins content were evaluated both for the blackberry methanolic extract, as well as the fruit itself.

### 2.2. Blackberry Fruit and Methanolic Extract Chemical Characterization

#### 2.2.1. Total Phenolic Content

Total phenolic content of blackberry fruit (triturated in liquid nitrogen) and its methanolic extract was determined by the Folin−Ciocalteu method (absorbance at 763 nm) [26], and the results were expressed as gallic acid equivalents/100 g sample, using a calibration curve from 4255 µg to 21,275 µg/250 µL of gallic acid. Although this method has some limitations, including overestimation of the results, we also quantified the main phenolic compound through LC-ESI-MS/MS.

#### 2.2.2. Identification and Quantification of Phenolic Compounds

LC-ESI-MS/MS analysis was performed for the identification of the flavonoids and ellagitannins in blackberry fruit and its methanolic extract, using a Prominence liquid chromatograph (Shimadzu, Tokyo, Japan) linked to an ion trap Esquire-LC mass spectrometer (Bruker Daltonics, Billerica, MA, USA), with an electrospray ionization (ESI) interface. The LC condition was a Prodigy 5 μm ODS3 column (250 × 4.60 mm; Phenomenex Ltd., Torrance, CA, USA), with a gradient of solvents consisting of (A) 0.5% formic acid in water and (B) 0.5% formic acid in acetonitrile, at flow rate of 0.75 mL/min, column temperature at 25 °C, and 5 µL injection. In the case of the fruit phenolic quantification, before injection, the extract was passed through a polyamide column (CC 6, Macherey-Nagel, Düren, Germany). On the other hand, the methanolic extract (for animal treatment) was directly injected. The ESI was used in the positive mode for anthocyanins and in the negative mode for the other classes of flavonoids. A full scan from *m*/*z* 100 to 1500 was carried out. The compounds were identified according to retention times of authentic standards, when possible, as well as by absorption spectrum similarity (525 nm for cyanidins; 270 nm for ellagic acid and quercetin), mass spectral characteristics, and comparison with the literature data [27].

#### 2.2.3. Total Antioxidant Capacity

The 2,2-Diphenyl-1-picrylhydrazyl (DPPH; 515 nm) [28] and oxygen radical absorbance capacity (ORAC; excitation wavelength at 540 nm and emission at 565 nm) [29] methods were used to analyze the antioxidant capacity of blackberry fruit and its methanolic extract. The results from the DPPH and ORAC were expressed as millimoles of Trolox equivalents/100 g sample.

### 2.3. Experimental Design

Male and female C57BL/6 mice (21 days old) were obtained from the colony of the Faculty of Pharmaceutical Sciences, University of São Paulo, Brazil, and were maintained under controlled conditions of temperature (22 ± 2 °C) and relative humidity (55 ± 10%) and light–dark periods of 12 h (light period from 6 a.m. to 6 p.m.), with free access to drinking water or water plus blackberry methanolic extract and to an AIN-93G (Harlan^®^, Madison, WI, USA) control diet. This study was approved by the Ethics Committee on Animal Experiments of the Faculty of Pharmaceutical Sciences, University of São Paulo (Protocol Number 411). The blackberry extract treatment was performed by diluting blackberry methanolic extract in the drinking water to attain a total phenolic compound concentration of 0.8 mg/mL.

**Figure 1:**
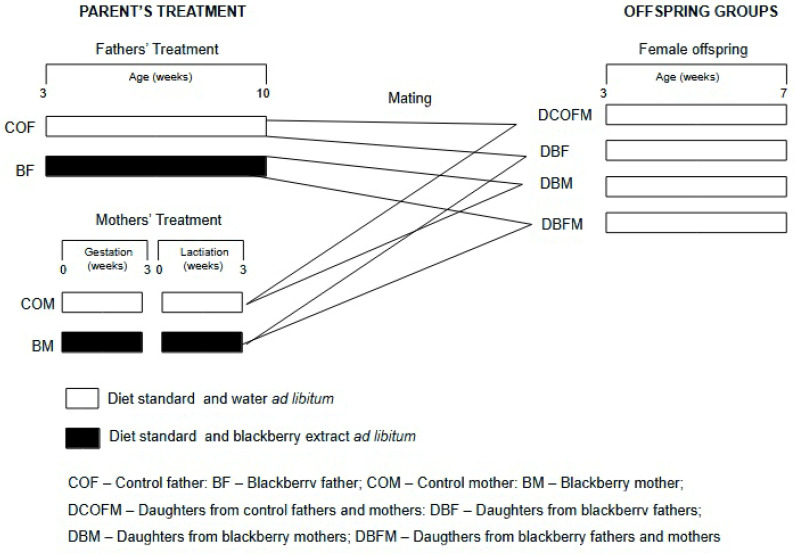
Experimental design of study.

Figure 1 shows the experimental design. A total of 24 males were distributed in 2 groups (*n* = 12/group): control fathers (COFs), who received drinking water, and blackberry fathers (BFs), who received the fruit methanolic extract dissolved in water. The treatments started when the males were 21 days old and lasted for 7 consecutive weeks, when they reached sexual maturation. The males were then mated with the females (1 male to 2 females). A total of 48 females were distributed in 2 groups (*n* = 24/group): control mothers (COMs), who received drinking water, and blackberry mothers (BMs), who received the fruit methanolic extract dissolved in water. The treatments lasted for 6 consecutive weeks and comprised the gestation and lactation stages. Thus, offspring were generated by the following matings: COF and COM (COF/COM); COF and BM (COF/BM); COM and BF (COM/BF); and BM and BF (BM/BF). Female offspring received a standard diet (Nuvital, Colombo, Brazil) and water ad libitum from weaning at 21 days of age until they were seven weeks old. All animals were euthanized by anesthetic overdose (isoflurane), and blood samples and organs were collected and maintained at −80 °C. Their body weight was recorded twice a week, and their food and drink consumption were evaluated four times a week.

### 2.4. Father’s Reproductive Parameters

#### 2.4.1. Tissue Collection and Preparation

After euthanasia, the left testis and epididymis were immediately weighed and fixed in methacarn solution (methanol, chloroform, and glacial acetic acid, 6.0:3.0:1.0, *v*/*v*) for 24 h. The relative weight (absolute weight/total body weight × 100) was determined. The Paraplast^®^-embedded testis were sectioned at 3–5 μm-thickness and stained with hematoxylin and eosin.

#### 2.4.2. Testicular Histomorphometrical Analysis

Percentages of the testis’s tubular and interstitial areas were determined by measuring the area occupied by the seminiferous tubules and interstitium, in fifteen fields per animal [30]. Thirty round tubules for each animal were selected randomly to measure the tubular diameter [30]. Analyses were performed using AxioVision 4.8 software coupled to a microscope at 200× magnification.

#### 2.4.3. Sperm Parameters

The right testis was stored at −20 °C and used to estimate the daily sperm production. After tissue homogenization in an STM solution (0.9% NaCl; 0.05% Triton X-100), spermatids in stages 14–16 were counted in Neubauer hemocytometer chambers (Optik Labor, Görlitz, Germany). The calculation was performed according to Thayer et al. [31], and the data were expressed as the number of sperm (×10^6^) per testis per day.

The sperm samples were obtained from the epididymis cauda in phosphate buffer saline solution by diffusion for 15 min. Then, 200 spermatozoa were analyzed per animal under a light microscope at 400× magnification and classified as normal or abnormal, in the text according to Seed et al. [32]. The abnormalities included head and tail alterations. The results were expressed as the percentage of normal sperm.

#### 2.4.4. Fathers’ Plasma Testosterone Levels

The fathers’ plasma testosterone levels were determined, in duplicate, by chemiluminescence using automated UniCel dxI 800 (Beckman Coulter^®^, Brea, CA, USA) equipment, with a sensitivity of 10 ng/dL.

### 2.5. Gestational Outcomes and Offspring Developmental Parameters

Gestational outcomes included the number of pregnant females after mating; the total number of pups per group; the number of pups per mother; and perinatal mortality. Offspring developmental parameters included body weight gain and relative organ weights.

### 2.6. Fathers, Mothers, and Female Offspring Antioxidant Capacity and Enzyme Activity

The plasma antioxidant capacity of the mothers, fathers, and female offspring was evaluated using the ORAC method [19]. For antioxidant enzyme activity quantification, liver/and or testis samples from the fathers, mothers, and female offspring were processed in potassium phosphate buffer (0.1 M, pH 7.0). The obtained extract was centrifuged at 10,000 rpm at 4 °C for 15 min. The remaining pellet was used to analyze superoxide dismutase (sod) [33]; catalase (cat) [34]; and gluthatione peroxidase (gpx) [35] enzymatic activities through spectrophotometry. Analyses were performed in triplicate, and the results were adjusted according to protein content [36].

### 2.7. Female Offspring Glucose Tolerance

For the intraperitoneal glucose tolerance test for the fathers, mothers, and daughters, the animals were fasted overnight. The animals received a dextrose solution i.p. (2 g/kg body weight), and glycemia was determined via caudal puncture after 15, 30, 60, 90, and 120 min, through a portable glycosometer (AccuCheck Performa Nano, Roche, São Paulo, Brazil) [37].

### 2.8. Statistical Analysis

The statistical analysis was conducted with GraphPad Prism 9.0 (GraphPad Software Inc., La Jolla, CA, USA). All data were tested for normality. Student’s *t*-test was applied to evaluate blackberry extract consumption by the fathers or mothers. To evaluate the impact of maternal and paternal blackberry extract consumption on the female offspring, an ANOVA model for two fixed factors (father and mother blackberry consumption) and the multiple comparisons method of Tukey were used. The Chi-square test was further used when proportions were analyzed in the female offspring. The level of statistical significance was set at *p* ≤ 0.05. The data are presented as the mean and standard error of the mean (SEM).

## 3. Results

### 3.1. Blackberry Fruit and Methanolic Extract Chemical Characterization

The chemical analysis of blackberry fruit showed that the most prevalent polyphenolic compounds were ellagitannins, followed by anthocyanins (mostly cyanidin-3-glucoside) and quercetin (Table 1 and Supplementary Table S1). In the methanolic blackberry extract, such composition was altered, showing a higher proportion of cyanidins, followed by ellagitannins and quercetin. This lower concentration of ellagitannins in the blackberry extract could be due to fruit processing during polyphenolic compounds enrichment extraction, since ellagitannins are found mostly in the seeds, which are removed after filtration during the extract production. The antioxidant capacity of blackberry fruit was higher than its methanolic extract (Table 1).

### 3.2. Fathers and Mothers

#### 3.2.1. Body Mass Gain and Organ Weight

There was no difference (*p* > 0.05) between the males from the COF and BF groups regarding food consumption and body mass gain during preconception, as well as the relative weights of the testis, epididymis, liver, lung, heart, kidney, and adipose tissues (Table 2). Similarly, there was no difference (*p* > 0.05) between the mothers from the COM and BM groups regarding food consumption and body mass gain during gestation and lactation, as well as the relative weights of the uterus, liver, lung, heart, and kidney (Table 3).

#### 3.2.2. Antioxidant Capacity and Enzymatic Activity

Compared to the fathers from the COF group, the fathers from the BF group presented increased (*p* ≤ 0.05) plasma antioxidant capacity, while there were no differences (*p* > 0.05) between the mothers from the COM and BM groups regarding this parameter (Table 4). Compared to the fathers from the COF and the mothers from the COM groups, respectively, the fathers from the BF and the mothers from the BM groups presented increased (*p* ≤ 0.05) and decreased (*p* ≤ 0.05) hepatic SOD activity (Table 4). There was no difference (*p* > 0.05) between the fathers from the COF and BF groups and between the mothers from the COM and BM groups regarding hepatic CAT and GPx activity (Table 4). Compared to the fathers from the COF group, the fathers from the BF group presented decreased (*p* ≤ 0.05) testicular SOD and CAT activity, while there was no difference (*p* > 0.05) regarding testicular GPx activity (Table 4).

#### 3.2.3. Reproductive Parameters and Litter Characteristics

**Figure 2:**
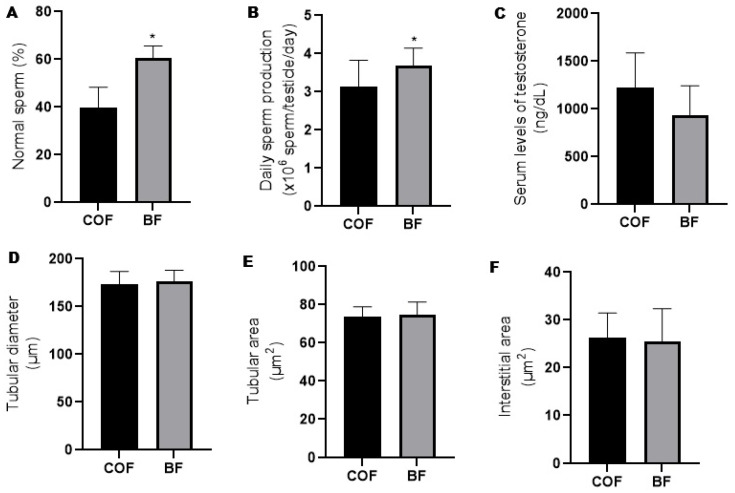
Reproductive parameters of male mice (fathers) from COF and BF groups. (**A**): Sperm morphology (*n* = 10/group); (**B**): daily sperm production (*n* = 11/group); (**C**): serum levels of testosterone (*n*= 4/group); (**D**): tubular diameter of seminiferous tubule (*n* = 6/group); (**E**): tubular area of seminiferous tubule (*n* = 6/group); and (**F**): interstitial area of seminiferous tubule (*n* = 6/group). * Statistically significant difference (*p* ≤ 0.05) when compared to COF group, according to Student’s *t*-test. COFs: control fathers, BFs: blackberry fathers.

Compared to the fathers from the COF group, the fathers from the BF group presented increased (*p* ≤ 0.05) prevalence of normal sperm morphology (Figure 2A) and daily sperm production (Figure 2B). There was no difference (*p* > 0.05) between the fathers from the COF and BF groups regarding serum levels of testosterone (Figure 2C), seminiferous tubular diameter (Figure 2D), and tubular (Figure 2E) and interstitial area (Figure 2F).

Compared to the COFM group, the BF group presented a tendency of increased (*p* = 0.067) pregnancy rate, while there were no differences (*p* > 0.05) among the COFM, BM, and BFM groups regarding this parameter (Table 5). Compared to the COFM group, the BF, BM, and BFM groups presented decreased (*p ≤* 0.05) perinatal mortality. Compared to the BF and BM groups, the BFM group presented decreased (*p ≤* 0.05) perinatal mortality. There was no difference (*p* > 0.05) among all the groups regarding the number of total pups/litter (Table 5).

### 3.3. Female Offspring

#### 3.3.1. Body Weight at Weaning, Body Mass Gain, and Organ Weight

Compared to the DCOFM, DBF, and DBFM groups, the DBM group presented decreased (*p* ≤ 0.05) body weight at weaning, while there were no differences (*p* > 0.05) among the DCOFM, DBF, and DBFM groups regarding this parameter (Table 6). There was no difference (*p* > 0.05) among all the groups regarding body mass gain and the relative weights of the uterus, liver, lung, heart, kidney, and retroperitoneal adipose tissue (Table 6).

#### 3.3.2. Antioxidant Capacity and Enzymatic Activity

**Figure 3:**
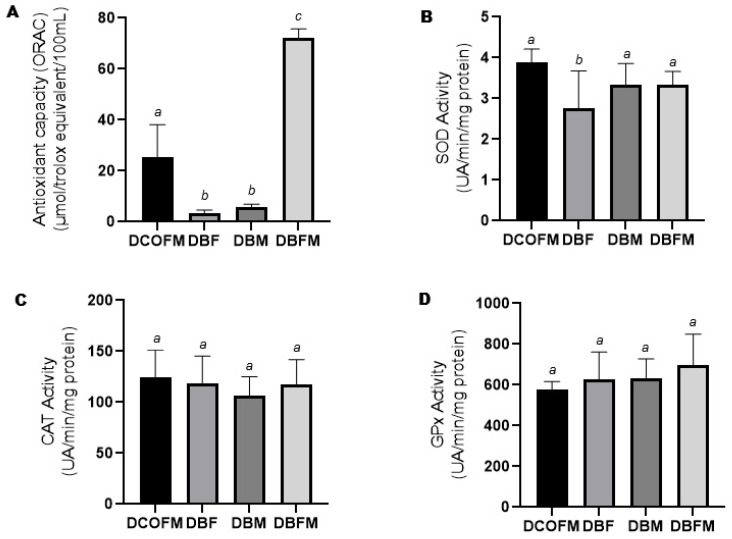
Plasma antioxidant capacity and liver antioxidant enzyme activity of 7-week-old animals from DCOFM, DBF, DBM, and DBFM groups. (**A**): Plasma antioxidant capacity of female offspring (*n* = 5); (**B**): liver SOD activity of female offspring (*n* = 6/group); (**C**): liver CAT activity of female offspring (*n* = 6/group); and (**D**): liver GPx activity of female offspring. Different letters between groups indicate statistically significant differences (*p* ≤ 0.05), according to analysis of variance with two fixed factors and multiple comparisons method of Tukey. DCOFMs: daughters control fathers and mothers; DBFs: daughters blackberry fathers; DBMs: daughters blackberry mothers; DBFMs: daughters blackberry fathers and mothers.

Compared to the DCOFM group, the DBF and DBM groups presented decreased (*p* ≤ 0.05), while the DBFM group presented increased (*p* ≤ 0.05) plasma antioxidant capacity (Figure 3A). Compared to the DBF and DBM groups, the DBFM group presented increased (*p* ≤ 0.05) plasma antioxidant capacity (Figure 3A). There was no difference (*p* > 0.05) between the DBF and DBM groups regarding this parameter. Compared to the DCOFM group, the DBF group presented decreased (*p* ≤ 0.05) hepatic SOD activity, while the DBM and DBFM groups did not present differences (*p* > 0.05) regarding this parameter (Figure 3B). There was no difference (*p* > 0.05) among all the groups regarding hepatic CAT and GPx activity (Figure 3C and Figure 3D, respectively).

#### 3.3.3. Intraperitoneal Glucose Tolerance

Compared to the DCOFM group (268 ± 38 mg/dL), the DBM (246 ± 32 mg/dL) and DBFM (232 ± 21 mg/dL) groups presented decreased (*p* ≤ 0.05) plasma glucose peak levels, while the DBF group (260 ± 24 mg) had no present difference (*p* > 0.05) regarding this parameter. There was no difference (*p* > 0.05) between the DBM and DBFM groups regarding this parameter.

## 4. Discussion

The blackberry fruit methanolic extract investigated in our study showed high anthocyanin content, represented mainly by cyanidin-3-glucoside, followed by ellagitannins and quercetin. These results are in line with previous studies showing that cyanidin comprises about 80–90% of total anthocyanins in different blackberry cultivars [38], and that blackberry contains a significant concentration of ellagitannins, and a lower concentration of quercetin. This latter polyphenol is considered the most abundant flavonol in this berry [1].

Blackberry anthocyanins’ metabolic effects have been studied in mice at levels of ingestion of 29 mg/kg body weight/day, which represents the consumption of 145 mg of anthocyanins (1–2 cups of fresh blackberry) in an average human [39,40]. In our study, the mice’s daily intake of blackberry anthocyanins was 244 ± 8 mg/kg/day, 319 ± 60 mg/kg/day, and 488 ± 98 mg/kg/day at the end of male preconception, gestation, and lactation, respectively. Thus, the level of anthocyanin ingestion in our study would be considered supranutritional. Importantly, despite this higher intake level, blackberry polyphenolic compounds did not induce apparent toxicological effects on both fathers and mothers, as no alterations in food intake, body weight gain, and organ weights were reported.

In our study, blackberry polyphenolic extract consumption increased plasma antioxidant capacity in male mice (fathers). Similar results were observed in male rats after the ingestion of a blackberry anthocyanin-enriched fraction [41]. In addition, we observed that the blackberry methanolic extract increased liver SOD activity, while it decreased testicular SOD and CAT activities in the male mice. These decreases could be a reflection of the increased plasma antioxidant capacity. In young rats, cocoa polyphenol supplementation increased liver total antioxidant capacity but not antioxidant enzyme activity [42]. This suggests that when the total antioxidant capacity of the organism is increased, the activity of antioxidant enzymes is less needed in order to maintain redox homeostasis. Contrary to our results, blackberry juice intake by the male rats did not alter SOD and CAT enzymes activities [43]. These differences could be due to variations in polyphenolic compound ingestion and species response.

Increased interest has been directed towards the role of dietary polyphenols in male reproductive physiology [44]. A polyphenol-enriched blueberry extract reversed oxidative stress induced by hypobaric hypoxia in the epididymis of Sprague–Dawley rats [45]. In addition, cyanidin-3-glucoside (250 and 500 mg/kg, but not 1000 mg/kg diet) protected against 3-chloro-1,2-propanediol-induced testicular damage and increased spermatogenesis in rats [46]. Importantly, these authors also showed that when consumed alone (500 mg/kg diet; dose equivalent to 300 mg in a 60 kg individual), this dietary anthocyanin did not exert toxic effects on the testis and sperm. Similarly, in our study, blackberry polyphenol extract consumption, in the absence of stressors, increased sperm quality and production, while no alterations in serum testosterone levels, seminiferous tubule structure, and testicular and epididymal relative weights were found. Of note, the blackberry methanolic extract also increased pregnancy rates. These effects could be related to the increased antioxidant status. Although low levels of ROS are critical for normal sperm physiology, including fertilizing capacity (acrosome reaction, hyperactivation, capacitation, and chemotaxis) and sperm motility, increased oxidative stress is associated with sperm DNA damage/apoptosis and lipid peroxidation, leading to subfertility and infertility [47]. This is the rationale for proposing redox modulating agents for use in the context of male reproduction [48]. To the best of our knowledge, this is the first report showing beneficial effects of blackberry polyphenols on the male reproductive system and fertility. This is relevant based on current estimations that male factors account for 30–50% of cases of infertility [49]. In addition, data from Jiang et al. [46], and ours, have shown an absence of male reproductive toxic effects (no alterations in testosterone levels, sperm morphology and production, and testicular morphology) from berries polyphenols reinforce their apparent safety, as supplementation with these bioactive compounds has been clinically considered for improving male fertility [50].

Contrary to what was observed for the paternal intervention with the blackberry polyphenols, no effects were observed on the mothers’ plasma antioxidant status and hepatic antioxidant enzymes. Similarly, no effects on oxidative stress and antioxidant capacity were observed after oligomeric grape seed proanthocyanidins supplementation in normal pregnant Kunming mice, while the hypertensive ones benefited [51]. Obese pregnant women supplemented with whole blueberry (2 cups/day) and soluble fibers showed reduced oxidative stress and increased serum total antioxidant capacity, but no alterations in serum GPX and SOD activities [52]. Altogether, these results suggest that pregnant women at high metabolic risk would particularly benefit from higher dietary polyphenol consumption. In addition, because some polyphenols, including resveratrol, have been considered for increased female fertility [53], it would be important to specifically investigate blackberry polyphenols’ effects on female fertility by providing them during the preconception stage under our experimental conditions.

Increased consumption of fruits and vegetables by both parents during preconception and gestation is recommended for promoting offspring health [54]. We observed that combined parental blackberry polyphenolic extract consumption led to a higher reduction in perinatal mortality compared to either paternal or maternal consumption. In addition, no effects on adult female offspring weight gain and organ weights were observed after all parental blackberry extract interventions. Quercetin consumption (50 mg/kg body weight) by pregnant rats did not alter placental morphology [55]. In addition, quercetin supplementation during gestation did not affect the mouse litter profile [56]. Similarly, blackberry juice consumption by rats during gestation did not alter the number of pups per litter [25], as we also observed in our study. From a developmental perspective, our data do not indicate apparent blackberry polyphenol toxicity to the conceptus. This is important since clinical and experimental data suggest that high polyphenol consumption, especially in late gestation, is associated with the constriction of the fetal ductus arteriosus [57].

It has been hypothesized that dietary intake of antioxidants during gestation could modify the in utero environment, leading to an antioxidant adaptation in the offspring, with the potential to prevent chronic diseases in adulthood [56]. Quercetin consumption (50 mg/kg body weight) by pregnant rats increased placental total antioxidant capacity, without affecting antioxidant enzymes [55]. In addition, a similar intervention with this polyphenol in female mice increased fetal hepatic antioxidant genes *Sod2* and *Nrf2* [56]. Adult offspring prenatally exposed to quercetin showed increased *Nrf2* hepatic expression, which was accompanied by decreased oxidative stress markers 8-oxo-dG and M1dG [56]. According to the authors, this sustained expression of the antioxidant gene could be due to epigenetic processes [58]. Quercetin further decreased endoplasmic reticulum stress and related inflammation programmed by maternal obesity in adult rat offspring [59]. On the other hand, in our study, paternal and maternal blackberry extract consumption similarly decreased the total plasma antioxidant capacity in the adult female offspring. Supplementation of rat dams with narigenin induced oxidative stress in the offspring’s cerebral tissue [60]. Some level of oxidative stress is physiologically needed during gestation, as reactive oxygen species act as biochemical signaling mediators for the proper development of the embryo/fetus [61]. Thus, interventions with redox homeostasis modulating compounds during this key developmental stage should be considered with great care, as an equilibrium between oxidant and antioxidant production is key during pregnancy, and its deregulation may pose a risk both to mother and offspring [61,62].

Interestingly, combined parental consumption of the blackberry polyphenolic extract had beneficial effects, reflected by increased antioxidant capacity in the female offspring. The reason for the different redox homeostasis responses, whether the blackberry intervention was through the father, mother, or both, is not clear. One potential explanation, which needs to be experimentally confirmed, would be that paternal and/or maternal consumption of these compounds distinctly affected the epigenetic status of redox homeostasis-related genes in the offspring. Both the male gametogenesis and embryonic/fetal stages involve important epigenetic remodeling necessary for fertilization and subsequent cell proliferation and differentiation [63]. Dietary polyphenols have been shown to affect DNA methylation, histone modifications, and microRNA expression in several model systems, although information on their epigenetic effects in early life is scarce [6,17]. Maternal hydroxytyrosol supplementation in a swine model of intrauterine growth restriction pregnancy increased fetal global DNA methylation [64]. Emerging data show that paternal and maternal interventions can induce different outcomes in the adult offspring. Importantly, opposing effects were observed in ovarian estrogen receptor beta expression, whether offspring were from obese fathers, mothers, or both [65]. Similarly, in humans, the increased level of glucorticoid receptor gene promoter methylation was observed in the offspring of the stress-exposed fathers, but not the mothers or both parents [66]. Accumulating data further suggest that paternal experiences can interact with maternal experiences, amplifying fetal metabolic programming effects [67]. Combined parental obesity/high-fat and high-sugar diet augments single-parent obesity effects on hypothalamus inflammation, leptin signaling, and obesity in adult mice [68] and rat offspring [69]. Maternal and paternal exercise have additive effects to improve glucose tolerance in mouse offspring as they age [70]. However, in our study, only maternal intervention with blackberry extract improved this metabolic parameter.

Parental interventions with polyphenols have also been shown to prevent complex phenotypes, such as breast cancer, in female offspring in animal models. Epigallocatechin-3-gallate-rich green tea polyphenol consumption by fathers during preconception inhibited breast tumor growth in transgenic female mice [71]. Blueberry exerted similar effects when provided to female transgenic mice during gestation and lactation [72]. Based on these data, it is important in further studies to evaluate whether parental consumption of blackberry polyphenol extract would exert similar breast cancer protective effects in descendants.

The fact that only one dose of blackberry polyphenolic compound was investigated is an important limitation in our study. Although few studies have systematically evaluated polyphenol toxic effects, data for blueberry anthocyanins suggest its safety (no alterations in organ weights and clinical biochemistry) at high levels of intake, with these polyphenols showing a NOAEL higher than 1000 mg/kg b.w./day (around 10 g polyphenol for a 70 kg human) in female rats [73]. The current literature suggests that the beneficial potential of polyphenol supplementation outweighs its potential harm in different conditions, such as non-alcoholic fatty liver disease [74] and polycystic ovarian syndrome [75]. However, the dual role of polyphenols, in which both protective effects against chronic disease and fetal adverse effects have been described, reinforces the need for careful consideration of maternal consumption of these bioactive compounds, especially in the third trimester [76]. Increased in utero exposure to dietary polyphenols could be associated with fetal ductal constriction, accompanied by higher ductal velocities and lower pulsatility indexes, as well as larger right ventricles [76]. Although the exact mechanisms are not clear, the inhibition of maternal prostaglandin E2 plasma levels by dietary polyphenols has been highlighted [77]. As there is still no consensus on the validity of polyphenol supplementation during pregnancy (11), the most prudent approach would be to offer these compounds to mothers through the consumption of plant-based diets [78]. Thus, it would be important to expand our experimental design in future studies to include a dose-dependent approach, adding both nutritional and pharmacological levels, to better understand blackberry polyphenolic compounds’ toxicological profile and influence on parental reproductive health, and metabolic and antioxidant programming in the offspring.

Although long-lasting developmental programming effects have been described after maternal or paternal exposure to metabolic stressors, such as obesity, protective effects of polyphenols in the DOHaD/POHaD context have been predominantly shown in young adult offspring [6,17]. Thus, it is important that future studies also consider the long-lasting effects of early life intervention with polyphenols. In addition, the impact of such interventions depends on the mothers’ dietary patterns. Resveratrol’s beneficial metabolic effects were shown in the offspring of high- but not low-fat-consuming pregnant/lactating rats [79]. In addition, such effects were more pronounced in female offspring [79]. Because of this, we opted to initially focus on the female offspring in our study, although it would also be important to extend our study to verify if there is a sex-dependent offspring response to maternal and/or paternal blackberry polyphenol intervention. Future studies with these polyphenols should also be conducted with obese mothers and fathers, and specifically provide them during maternal preconception, gestation, or lactation, as programming outcomes are profoundly influenced by the critical developmental window in which the intervention is set [6].

## 5. Conclusions

Altogether, our study advances the knowledge on the role of blackberry polyphenols in early life. All parental blackberry polyphenol interventions decreased offspring perinatal mortality, with combined fathers’ and mothers’ polyphenol consumption exerting the most pronounced effects. Paternal or maternal polyphenol consumption decreased the plasma total antioxidant capacity in the female offspring. On the other hand, combined parental consumption had opposing effects on the offspring. Only maternal polyphenol interventions increased glucose tolerance in the female offspring. These data only partially confirm our hypothesis that a combined paternal and maternal polyphenols intervention would lead to better outcomes in the offspring. To the best of our knowledge, this is the first study to show that blackberry polyphenol supplementation during paternal preconception and/or maternal gestation/lactation differentially affects paternal fertility and female offspring antioxidant status and glucose tolerance. Importantly, interactions were observed between the paternal and maternal consumption of these food bioactive compounds. This suggests that in order to promote long-term health in the descendants, nutritional interventions, including those with polyphenols, should target not only the mother but also the future father. Before this innovative strategy becomes a reality, clinical studies should be expanded to confirm this possibility.

## Figures and Tables

**Table 1:** Characterization of blackberry fruit and its methanolic extract.

	Blackberry Fruit	Blackberry Methanolic Extract
Total phenolic compounds (mg Gallic acid equivalent/100 g or 100 mL)	157 ± 23	154 ± 11
Cyanidin (mg/100 g or 100 mL)	105 ± 6	176 ± 2.4
Quercetin (mg/100 g or 100 mL)	26 ± 2	26 ± 1
Free ellagic acid (mg/100 g or 100 mL)	6 ± 0.2	8 ± 2
Total ellagic acid (ellagitannins) (mg/100 g or 100 mL)	430 ± 27	65 ± 47
Antioxidant capacity (DPPH) (µmoL Trolox equivalent/100 g or 100 mL)	1525 ± 88	80 ± 5
Antioxidant capacity (ORAC) (µmoL Trolox equivalent/100 g or 100 mL)	2265 ± 612	644 ± 770

The data are expressed as the mean ± standard deviation. All analyses were performed in triplicate.

**Table 2:** Body mass gain during preconception and relative organ weight of male mice (fathers) from the COF and BF groups.

Variables	Experimental Groups	
	COFs	BFs
Body mass gain (g)	19.25 ± 2.52	18.76 ± 2.45
Testis relative weight (%)	0.24 ± 0.07	0.25 ± 0.09
Epididymis relative weight (%)	0.08 ± 0.05	0.09 ± 0.04
Liver relative weight (%)	4.04 ± 0.36	4.14 ± 0.51
Lung relative weight (%)	0.76 ± 0.10	0.74 ± 0.10
Heart relative weight (%)	0.60 ± 0.07	0.59 ± 0.08
Kidney relative weight (%)	0.58 ± 0.05	0.60 ± 0.07
Abdominal adipose tissue relative weight (%)	1.85 ± 0.61	1.78 ± 0.63
Retroperitoneal adipose tissue relative weight (%)	0.60 ± 0.68	0.51 ± 0.29
Retroepididymal adipose tissue relative weight (%)	1.58 ± 0.74	1.53 ± 0.73

The data are expressed as the mean ± standard deviation. *n* = 12/group. There was no (*p* > 0.05) statistical difference between the groups, according to Student’s *t*-test. COFs: control fathers, BFs: blackberry fathers.

**Table 3:** Body mass gain during gestation and lactation, and relative organ weight of female mice (mothers) from the COM and BM groups.

Variables	Experimental Groups	
	COMs	BMs
Body mass gestational gain (g)	15.73 ± 1.67	15.89 ± 2.95
Body mass lactational gain (g)	−1.50 ± 1.61	−1.25 ± 1.21
Uterus relative weight (with ovary) (%)	0.46 ± 0.15	0.51 ± 0.12
Liver relative weight (%)	5.61 ± 1.37	5.20 ± 0.78
Lung relative weight (%)	0.97 ± 0.20	0.99 ± 0.09
Heart relative weight (%)	0.68 ± 0.14	0.77 ± 0.19
Kidney relative weight (%)	0.54 ± 0.08	0.61 ± 0.07

The data are expressed as the mean ± standard deviation. *n* = 12/group. There was no (*p* > 0.05) statistical difference between the groups, according to Student’s *t*-test. COMs: control mothers, BMs: blackberry mothers.

**Table 4:** Plasma antioxidant capacity and liver and testis antioxidant enzymatic activity of male (fathers) or female (mothers) mice from COF, BF, COM, and BM groups.

Parameters	Experimental Groups			
	COFs	BFs	COMs	BMs
Plasma antioxidant capacity (ORAC) (µmoL Trolox equivalent/100 mL)	8.19 ± 3.20	58.60 ± 9.64 *	80.33 ± 20.41	80.74 ± 10.06
SOD activity (liver) (UA/min/mg protein)	2.27 ± 0.23	4.76 ± 0.31 *	3.96 ± 0.93	2.88 ± 0.45 *
CAT activity (liver) (UA/min/mg protein)	136.40 ± 14.58	151.76 ± 18.03	125.69 ± 18.20	129.57 ± 26.66
GPx activity (liver) (UA/min/mg protein)	514.39 ± 56.61	586.55 ± 95.28	620.86 ± 127.59	511.27 ± 106.46
SOD activity (testis) (UA/min/mg protein)	28.16 ± 0.37	17.43 ± 1.16 *	-	-
CAT activity (testis) (UA/min/mg protein)	20.25 ± 4.86	9.83 ± 2.36 *	-	-
GPx activity (testis) (UA/min/mg protein)	43.05 ± 5.78	44.32 ± 7.89	-	-

The data are expressed as the mean ± standard deviation. *n* = 6/group. * Statistically significant difference (*p* ≤ 0.05) when compared to the COF or COM groups, according to Student’s *t*-test. COFs: control fathers, BFs: blackberry fathers, COMs: control mothers, BMs: blackberry mothers.

**Table 5:** Reproductive parameters of mice from COFM, BF, BM, and BFM groups.

Parameters	Experimental Groups			
	COFMs	BFs	BMs	BFMs
Pregnancy rate (%)	63 ^a^	79 ^b^	71 ^a^	75 ^a^
Perinatal mortality (%)	34 ^a^	24 ^b^	19 ^b^	9 ^c^
Number of total pups/litter	7 ± 1 ^a^	7 ± 2 ^a^	7 ± 2 ^a^	7 ± 2 ^a^

The data are expressed as the mean ± standard deviation. *n* = 12 matings/group. The different letters between the columns indicate a statistically significant difference (*p* ≤ 0.05), according to the Chi-square test (pregnancy rate or perinatal mortality) or analysis of variance with two fixed factors and the multiple comparisons method of Tukey (number of total pups/litter). COFMs: control fathers and mothers; BFs: blackberry fathers; BMs: blackberry mothers; BFMs: blackberry fathers and mothers.

**Table 6:** Body weight and relative organ weight of 7-week-old animals from DCOFM, DBF, DBM, and DBFM groups.

Variables	Experimental Groups			
	DCOFMs	DBFs	DBMs	DBFMs
Body weight at weaning (g)	9.05 ± 1.23 ^a^	8.91 ± 1.83 ^a^	7.67 ± 1.28 ^b^	9.02 ± 1.23 ^a^
Body mass gain (g)	7.17 ± 1.51 ^a^	6.98 ± 1.79 ^a^	7.63 ± 2.28 ^a^	7.05 ± 1.58 ^a^
Uterus relative weight (with ovary) (%)	0.66 ± 0.21 ^a^	0.54 ± 0.22 ^a^	0.51 ± 0.14 ^a^	0.68 ± 0.23 ^a^
Liver relative weight (%)	0.66 ± 0.07 ^a^	0.62 ± 0.08 ^a^	0.70 ± 0.09 ^a^	0.69 ± 0.11 ^a^
Lung relative weight (%)	0.84 ± 0.07 ^a^	0.71 ± 0.11 ^a^	0.79 ± 0.09 ^a^	0.78 ± 0.08 ^a^
Heart relative weight (%)	5.77 ± 0.57 ^a^	5.00 ± 1.10 ^a^	5.64 ± 0.27 ^a^	5.23 ± 0.36 ^a^
Kidney relative weight (%)	0.68 ± 0.07 ^a^	0.63 ± 0.08 ^a^	0.70 ± 0.06 ^a^	0.66 ± 0.08 ^a^
Retroperitoneal adipose tissue relative weight (%)	0.67 ± 0.29 ^a^	0.61 ± 0.28 ^a^	0.53 ± 0.19 ^a^	0.52 ± 0.14 ^a^

The data are expressed as the mean ± standard deviation. *n* = 15 (DCOFMs), 20 (DBFs), *n* = 9 (DBMs), and *n* = 18 (DBFM)s. The different letters between the columns indicate statistically significant differences (*p* ≤ 0.05), according to analysis of variance with two fixed factors and the multiple comparisons method of Tukey. DCOFMs: daughters control fathers and mothers; DBFs: daughters blackberry fathers; DBMs: daughters blackberry mothers; DBFMs: daughters blackberry fathers and mothers.

## Data Availability

Data is contained within the article and Supplementary Materials.

## Supplementary Materials

The following supporting information can be downloaded at: https://www.mdpi.com/article/10.3390/antiox14070779/s1, Table S1: Mass spectra of flavonoids from Blackberry extract.
