# Supplementary material for: Paternal and/or Maternal Blackberry (Rubus spp.) Polyphenolic Extract Consumption Improved Paternal Fertility and Differentially Affected Female Offspring Antioxidant Capacity and Metabolic Programming in a Mouse Model
Source: Antioxidants (Basel). 2025 Jun 25;14(7):779. doi: 10.3390/antiox14070779 (PMC12291980; doi:10.3390/antiox14070779)
Supplement: Supplementary file 1 [file antioxidants-14-00779-s001.zip › antioxidants-3603029-supplementary.pdf]

Supplementary Table S1. Mass spectra of flavonoids from Blackberry extract.

RT (min)	MS (m/z)	MS2 (m/z)	Proposed compounds
Positive mode			
10	449	287	Cyanidin 3-glucoside(*)
10.9	595	449; 287	Cyanidin 3-rutinoside(*)
15.2	593	287	Cyanidin glycoside
19.2	465	303; 285; 229; 165	Quercetin 3-glucoside(*)
14.5	535	449; 287	Cyanidin 3-(6″-malonyl-glucoside)
Negative mode			
16.9	477	301; 179; 151	Quercetin glucuronide-1
19.8	477	301; 179; 151	Quercetin glucuronide-2
20.9	609	463; 301; 151	Quercetin 3-rutinoside(*)
22.9	461	285	Kaempferol glucuronide

RT: Retention time; (*) Identity confirmed using standard.
